# CRISPR/Cas9 Based Site-Specific Modification of FAD2 *cis*-Regulatory Motifs in Peanut (*Arachis hypogaea L*)

**DOI:** 10.3389/fgene.2022.849961

**Published:** 2022-04-27

**Authors:** Anjanasree K. Neelakandan, David A. Wright, Sy M. Traore, Xiangyu Chen, Martin H. Spalding, Guohao He

**Affiliations:** ^1^ Department of Genetics, Development and Cell Biology, Iowa State University, Ames, IA, United States; ^2^ Department of Agricultural and Environmental Sciences, Tuskegee University, Tuskegee, AL, United States; ^3^ Crops Research Institute, Fujian Academy of Agricultural Sciences, Fuzhou, China

**Keywords:** regulatory element, gene editing, agrobacterium-mediated transformation, fatty acid, peanut

## Abstract

Peanut (*Arachis hypogaea* L.) seed is a rich source of edible oil, comprised primarily of monounsaturated oleic acid and polyunsaturated linoleic acid, accounting for 80% of its fatty acid repertoire. The conversion of oleic acid to linoleic acid, catalyzed by Fatty Acid Desaturase 2 (FAD2) enzymes, is an important regulatory point linked to improved abiotic stress responses while the ratio of these components is a significant determinant of commercial oil quality. Specifically, oleic acid has better oxidative stability leading to longer shelf life and better taste qualities while also providing nutritional based health benefits. Naturally occurring *FAD2* gene knockouts that lead to high oleic acid levels improve oil quality at the potential expense of plant health though. We undertook a CRISPR/Cas9 based site-specific genome modification approach designed to downregulate the expression of two homeologous *FAD2* genes in seed while maintaining regulation in other plant tissues. Two *cis*-regulatory elements the RY repeat motif and 2S seed protein motif in the 5′UTR and associated intron of *FAD2* genes are potentially important for regulating seed-specific gene expression. Using hairy root and stable germ line transformation, differential editing efficiencies were observed at both CREs when targeted by single gRNAs using two different gRNA scaffolds. The editing efficiencies also differed when two gRNAs were expressed simultaneously. Additionally, stably transformed seed exhibited an increase in oleic acid levels relative to wild type. Taken together, the results demonstrate the immense potential of CRISPR/Cas9 based approaches to achieve high frequency targeted edits in regulatory sequences for the generation of novel transcriptional alleles, which may lead to fine tuning of gene expression and functional genomic studies in peanut.

## Introduction

Peanut (*Arachis hypogaea* L.) or groundnut is an important legume crop due to the seed being a rich source of edible oil, protein and fiber. The oil content is about 50%, of which oleic and linoleic acids account for 80% of the total fatty acid with the ratio of these components largely determining oil quality. Therefore, the conversion of monounsaturated oleic acid to polyunsaturated linoleic acid by the Fatty Acid Desaturase 2 (FAD2) enzyme is a key regulatory point. Peanut oil is naturally high in linoleic acid, making it prone to oxidation, which reduces oil stability leading to rancidity and poor flavor characteristics. However, linoleic acid is important for healthy plant growth and it is also an essential fatty acid for humans (Guan et al., 2012; Marangoni et al., 2020). High oleic acid is beneficial for improved shelf life through oxidative stability, improved flavor characteristics and consumption may lead to better cardiovascular health in humans (O’Byrne et al., 1997; Yu et al., 2008; Lim et al., 2017). The natural *FAD2* knockout mutant line, F435, is devoid of FAD2 enzyme activity in seed and accumulates 80% oleic acid as observed by Norden et al., 1987. On the surface, these mutations may seem optimal for oil quality, however, introgression of these mutations into other peanut varieties is time consuming and knockout mutations may leave plants vulnerable during periods of stress (Zhang et al., 2012). Therefore, an ideal strategy to engineer oil composition without compromising plant health may involve downregulation of *FAD2* gene expression in seed while minimizing pleiotropic effects in other vegetative tissues through promoter modification.

The *FAD2* gene family has been extensively characterized from many plant species including peanut (Janila et al., 2016; Dar et al., 2017). Cultivated peanut is an allotetraploid (AABB, 2n = 4x = 40) and the genome contains two functional homeologous genes, *AhFAD2A* and *AhFAD2B*, derived from the diploid ancestors *Arachis duranensis* and *Arachis ipaensis*, respectively. FAD2 is the key enzyme that is responsible for biosynthesis of polyunsaturated acid in non-photosynthetic tissues, such as roots and developing seeds in oilseed plants (Miquel and Browse, 1992), while some *FAD2* genes are targeted to stems and leaves (Cao et al., 2013; Lee et al., 2020). *FAD2* genes typically have a conserved single large intron in the 5′-untranslated region (UTR), which has been suggested to have enhancer activity in mediating transcriptional regulation. This has been shown in sesame (Kim et al., 2006), and *Brassica napus* (Xiao et al., 2014) by deletion mapping approaches and *Arabidopsis* and olives by gene association studies (Menard et al., 2017; Salimonti et al., 2020).

Seed development, including embryogenesis and seed maturation, is regulated by a combination of hormonal, genetic and metabolic controls (Gutierrez et al., 2007). Differing clusters of *cis*-regulatory elements (CREs) in the promoters of seed associated proteins are bound by various combinations of transcription factors (TFs) including LEAFY COTYLEDON1 (LEC1), ABSCISIC ACID INSENSITIVE3 (ABI3), FUSCA3 (FUS3), and LEC2 (Parcy et al., 1997; Boulard et al., 2017; Jo et al., 2019; Tang et al., 2021). These interactions lead to formation of tightly controlled regulatory pathways in seed and other tissues making CREs potentially useful targets for altered gene expression. Among the CREs, the RY repeat element (Bäumlein et al., 1992; Fujiwara and Beachy, 1994) and 2S seed protein motif (Stålberg et al., 1996) are crucial for regulatory activity in a number of seed-specific promoters (Reidt et al., 2000; Chatthai et al., 2004).

RNA programmable CRISPR/Cas nucleases trigger sequence-specific double stranded breaks (DSB)s that are subject to error prone non-homologous end joining (NHEJ) repair or template dependent, homology-directed repair (HDR) pathways. NHEJ, the most frequently used repair mechanism in plants, often leading to insertions or deletions (indels) at the target site. This technology is used to generate gene knockouts when coding regions are targeted, therefore, this approach has been widely used for plant genome editing applications (Manghwar et al., 2019).

Our overarching goal was to utilize CRISPR/Cas9-mediated gene editing to modify *cis*-regulatory elements in the 5′ UTR and intron of *FAD2* genes for functional characterization of these elements and to potentially generate seeds with increased oleic acid content without affecting the fatty acid composition in other plant tissues. We designed guide RNAs (gRNAs) specific to the RY repeat element and 2S seed protein motif of peanut *FAD2* genes then transformed peanut tissues to explore the potential of modifying transcriptional regulation of *FAD2* gene expression in seeds. Our findings demonstrated high frequency modification of FAD2 RY and 2S motifs by CRISPR/Cas9-mediated gene editing and alteration of the oil profile in seed.

## Materials and Methods

### FAD2 Gene Sequence Confirmation and Analysis

**FIGURE 1:**
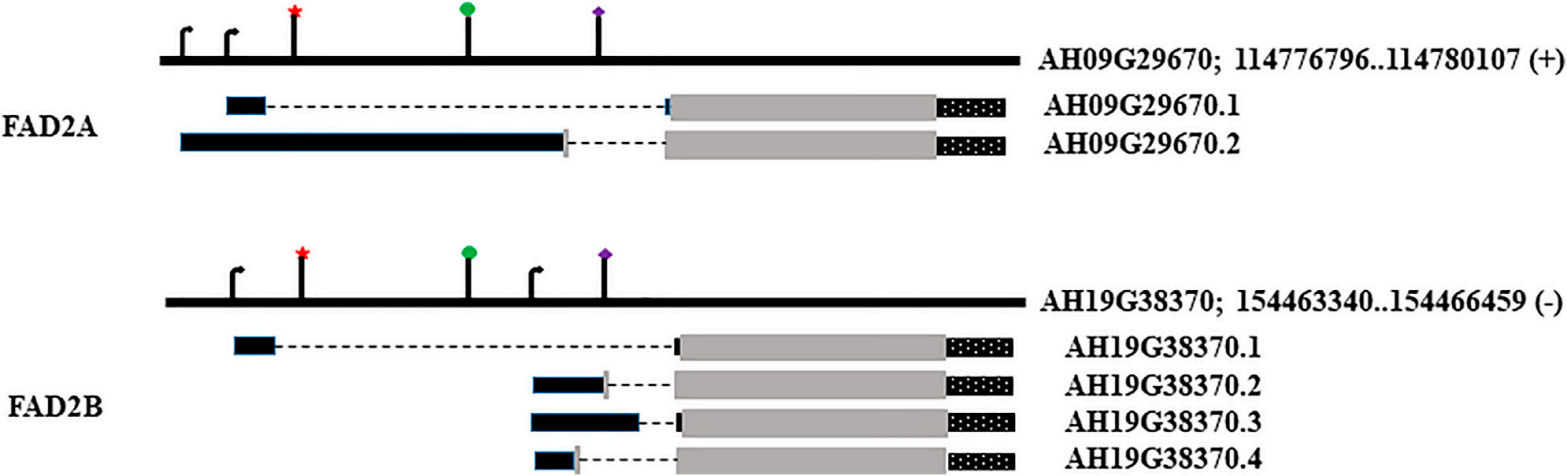
Genomic loci, transcript models and location of regulatory elements. Star denotes RY element; circle marks Initiator (INR) motif; diamond marks the 2S seed protein element. The arrows denote transcription start sites. Black rectangles denote 5′UTR, grey for coding sequence and dotted rectangles stand for 3′UTR, broken line represents the intron in the 5′UTR.

The *AhFAD2A* and *AhFAD2B* genes from genotype GT-C20 were characterized by PCR and sequence analysis to serve as a reference. The gene models for selected isoforms, FAD2A (AH09G29670) and FAD2B (AH19G38370) (Figure 1) and expression data were obtained from the Peanut Genome Resource (http://peanutgr.fafu.edu.cn/index.php; Sinha et al., 2020). The *cis*-elements that are present in the region upstream of the coding sequence were predicted using PLACE (https://www.dna.affrc.go.jp/PLACE/?action=newplace; Higo et al., 1999) and PlantCARE (http://bioinformatics.psb.ugent.be/webtools/plantcare/html/; Lescot et al., 2002).

### Construction of Initial Vectors

**FIGURE 2:**
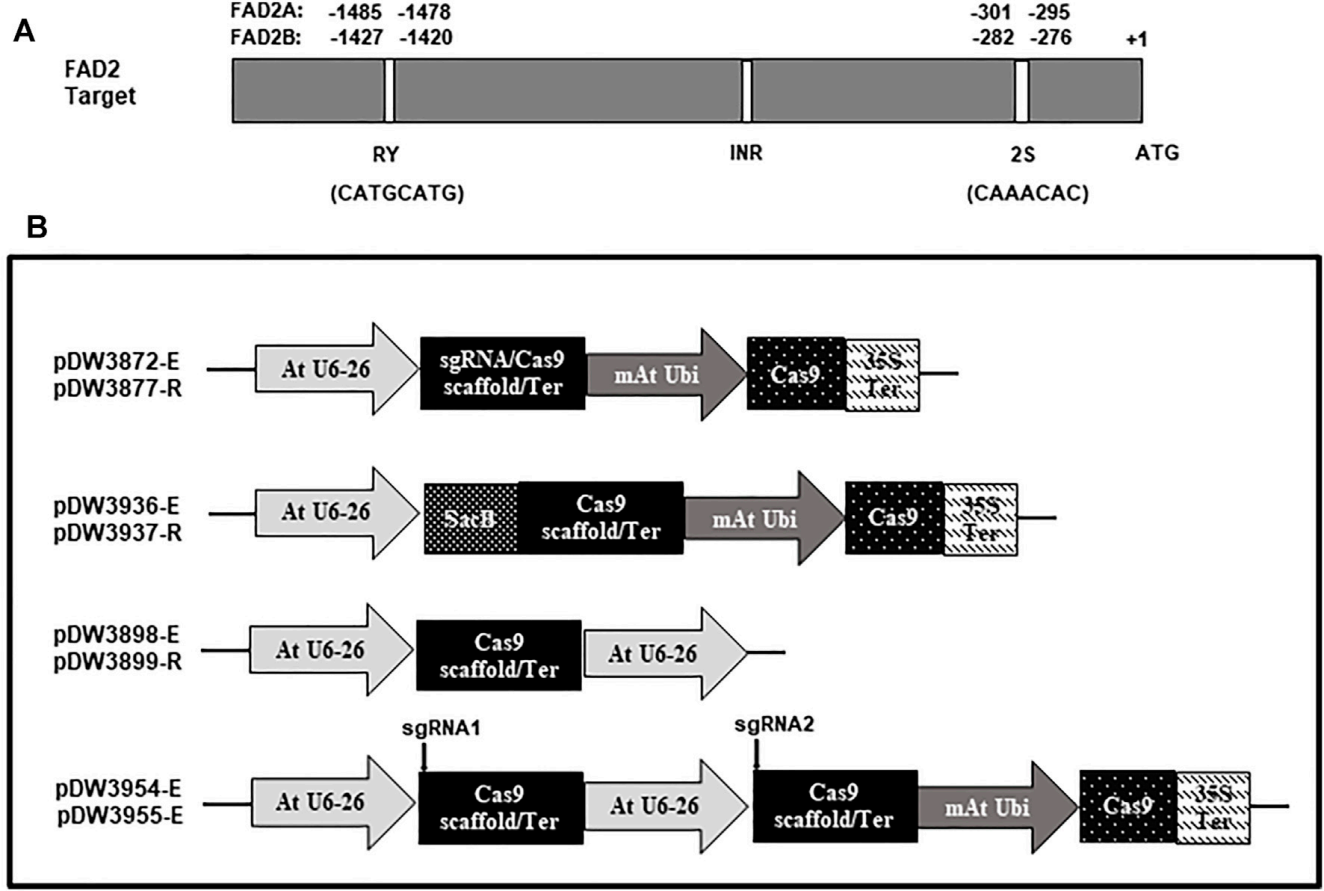
Schematic representation of the FAD2 targets and the vectors used in the study. **(A)**. Targets location and sequence in the promoter of FAD2A and FAD2B; **(B)**. Vectors with different scaffolds [Cas9-extended scaffold (-E) and Cas9-regular scaffold (-R)].

A dicot codon optimized Cas9 gene was excised from the vector pDW3602 as a *Bam*HI/*Spe*I fragment and ligated into pDW3868 using the same enzyme sites to generate pDW3872. This construct has an *Arabidopsis* U6 promoter from pTF101-AtCa9-GmRCA#1, dual *Bsa*I restriction sites for targeting oligonucleotide pair insertion, an extended gRNA scaffold (Dang. et al., 2015), modified *Arabidopsis* UBI promoter, Cas9 gene, a CaMV 35s terminator, and a CaMV 35s promoter expressing the *Bar* gene as a selectable marker. The extended gRNA scaffold was used to test for enhanced targeting efficiency compared to a standard gRNA scaffold. The vector pDW3877 has the same components as pDW3872, except a standard gRNA scaffold replaces the extended gRNA scaffold. The constructs are depicted in Figure 2.

### Final Vector Construction

gRNA targeting oligonucleotides specific to the predicted RY and 2S seed protein motif CREs were designed using the Cas-Designer tool (Park et al., 2015). The oligonucleotide pairs were synthesized at the Iowa State University DNA facility and were annealed after phosphorylation to generate sticky ends that correspond to the overhangs generated by *Bsa*I restriction digestion of the expression vectors pDW3872 or pDW3877. The sequences of the gRNAs are provided in Table 1. The ligated oligo and vector combinations were transformed into competent DH10B *E coli* cells, which were selected on LB plates supplemented with 30 μg/ml kanamycin and grown at 37°C overnight. DNA from individual clones was purified using the IBI plasmid purification kit then clones were verified by DNA sequencing.

**TABLE 1:** Details of target sites and gRNAs used in hairy root assay.

*Cis* Element	sgRNA Target Sequence (5′—3′)	Purpose
RY repeat element	GAT​AAC​ATC​AAC​ATG​CAT​GCT	Induction of indels
2S Seed protein element	GAT​TTG​AAT​GCC​ACA​TGT​GTT	Induction of indels

The cloning strategy for dual gRNA constructs was based on a one step Golden Gate ligation process. For this, a *sacB* gene was cloned into the *Bsa*I sites of pDW3872 and pDW3877 to confer sucrose sensitivity, generating pDW3936 and pDW3937, respectively (Figure 2) Supplementary plasmids containing a *Bsa*I site, extended or standard gRNA scaffold, an Arabidopsis U6 promoter and a second *Bsa*I site, in that order, were created and designated pDW3898 and pDW3899, respectively (Figure 2). Note that the *Bsa*I sites in the supplementary plasmids was positioned so the Type IIs enzyme will generate unique overhangs that cannot anneal to the Cas9 vector without an intervening oligo pair. Briefly, 625 ng of each plasmid was digested with 2 µL of Promega T4 ligase buffer and 1 μL of *Bsa*I enzyme in a total volume of 18 μL at 37°C for 3 h 600 pmol of each oligo was phosphorylated in a total volume of 30 µL then appropriate pairs were annealed in a total of 60 µL. Finally, the 18 µL plasmid digest, 1 µL of each oligo pair, 1 µL of 10 mM ATP and 1 µL Promega T4 ligase were mixed for a total ligation volume of 22 μLs. The ligations were subjected to one cycle 16°C for 20 min then 49 cycles of 37°C for 5 min and 16°C for 5 min followed by one cycle at 65C for 20 min, in a PCR machine, to denature the enzymes. 2.5 µL of each ligation was transformed as described above. Successful dual oligo ligations were identified by positive/negative selection on LB medium supplemented with 30 μg/ml kanamycin and 5% sucrose. Sequence confirmed single and dual gRNA constructs were mobilized into *Agrobacterium rhizogenes* strain K599 by electroporation and selected using LB medium supplemented with 50 μg/ml kanamycin and grown at 28°C for 2 days.

### Hairy Root Transformation

*Agrobacterium rhizogenes* is a plant pathogenic soil bacterium that induces the formation of adventitious roots, called hairy roots, at the site of infection. Hairy roots are unique in that they are predominantly non-chimeric and derive from a single *de novo* root meristem (Falasca et al., 2000). Stable transformation is laborious, time consuming and largely genotype dependent in peanuts. Therefore, the availability of rapid, reliable and quantifiable gene editing evaluation systems like hairy root assays help to select and advance functional target and gRNA combinations for later stable transformation efforts.

Genotype GT-C20 seeds were kindly provided by Dr. Baozhu Guo in USDA/ARS at Tifton, GA, which has no known natural mutations of the *FAD2* genes (Yuan et al., 2019). Peanut seeds were surface sterilized and incubated on germination medium for 1 week with 16-h photoperiod at 28°C. Hairy root transformation was performed on hypocotyl explants as described by Yuan et al. (2019) with slight modifications. The *Agrobacterium* culture was suspended in the infection medium at an optical density of 0.6 and incubated for 20 min with constant stirring. The explants were co-cultured for 3 days in the dark and transferred to hairy root induction medium after washing with an antibiotic solution containing timentin (300 mg/L). Explants were incubated under fluorescent lights at 28°C with a 16-h photoperiod. After 1.5–2 weeks, transformed roots were harvested then explants were sub-cultured and maintained for 4–6 weeks.

### Amplification and Sequencing of Targets

Hairy roots were harvested aseptically into sterile tubes and kept at –20°C for future processing. Root tissues were crushed in dilution buffer provided in the Phire Plant Direct PCR Kit (Thermo Fisher Scientific), and the liquid fraction was used for direct PCR without purification, as per the manufacturer’s instructions. The oligo sequences for PCR fragment generation and sequence analysis are given in Table 2. The amplified products were purified using the IBI PCR product purification kit then subjected to Sanger Sequencing at the Iowa State University DNA facility.

**TABLE 2:** The oligo sequences used for PCR and Sanger Sequencing.

Oligo Name	Oligo Sequence (5′—3′)	Purpose
AHY1006	GTC​CTC​AAA​TAG​CTC​GAC​TG	Forward primer to amplify FAD2A distal promoter
AHY1007	AGG​GCC​CAG​AAG​CAA​TTA​TGA​TAC	Reverse primer to amplify FAD2A distal promoter
AHY1078	TTG​AAG​CAA​AGG​GGT​GAG​GTT​TTC	Forward primer to amplify FAD2A proximal promoter
AHY1073	CAA​GTC​AAT​AAT​CAG​TAA​TCT​AAT​G	Reverse primer to amplify FAD2A proximal promoter
AHY1012	GAA​TGA​GGA​TGG​GGA​CCA​ATA​TTC	Forward primer to amplify FAD2B distal promoter
AHY1013	AGG​GCC​CAG​AAG​CAA​TTA​CTA​ATG	Reverse primer to amplify FAD2B distal promoter
AHY1079	GAA​GTA​AGG​GTT​GGT​GAA​GTT​TTC	Forward primer to amplify FAD2B proximal promoter
AHY1015	GCA​CTA​CTA​CAA​AGC​TAA​TGG​TTC	Reverse primer to amplify FAD2B proximal promoter
AHY1074	CCA​ATG​TGA​GTG​AGA​CAA​CAA​C	Sequencing primer for FAD2A & B distal promoter
AHY1097	CTG​GCT​CCA​AGT​CCA​AGC​AAT​A	Sequencing primer for FAD2A and B proximal promoter

### Calyx Tube Injection Transformation

To avoid time-consuming, genotype-dependent and often recalcitrant tissue culture and regeneration, we have developed an *Agrobacterium*-mediated calyx tube injection method to quickly obtain putatively edited peanut seeds. CRISPR/Cas9 constructs were mobilized into *Agrobacterium* strain GV3101 using a freeze and thaw methodology (An et al., 1988). Transformed *Agrobacterium* were grown on LB plates supplemented with 50 μg/ml kanamycin at 28°C and colonies were picked after 2–3 days’ growth, followed by colony PCR to confirm mobilization of the constructs. Transformation confirmed *Agrobacterium* colonies were grown in 10 ml liquid LB cultures with shaking overnight at 28°C and supplemented with 50 μg/ml kanamycin. Bacterial cells were pelleted using low speed centrifugation (4,000 rpm) and resuspended into 10 ml sterile inoculation media composed of ½ MS supplemented with 25 g/L sucrose and 200 μL/L Silwet L77. The OD_600_ was adjusted to 0.6 to 0.8 by addition of inoculation media. Besides GT-C20, two additional genotypes (AU18-46 and Exp27-1516) were also used to test the method of calyx tube injection. 0.2 ml of the *Agrobacterium* suspension carrying a CRISPR/Cas9 vector was injected into individual peanut plant calyx tubes. Inoculated flowers were labelled and derived seeds were harvested for analysis. DNAs were extracted from T_1_ plants for sequencing FAD2 targets.

### Fatty Acid Composition Analysis

The fatty acid content was measured for each of the harvested seeds from T_0_ and T_1_ generations using an Agilent 7890A gas chromatograph (GC) with a flame ionization detector (FID). Seeds were individually crushed. Ground seed material was extracted in 4.0 ml heptane (Fisher Scientific) and converted to fatty acid methyl easters (FAMEs) with 500 µL 0.5 N sodium methoxide (NaOCH_3_) in methanol. A fatty acid methyl ester (FAME) standard mix RM-3 plus four additional FAMES (Sigma) were mixed and used to establish peak retention times. Fatty acid composition was determined by identifying and calculating relative peak areas (Wang et al., 2009).

## Results

The *FAD2A* (AH09G29670) and *FAD2B* (AH19G38370) gene models used in this study have a 5′ UTR with a single intron, coding sequence, and a 3′ UTR as shown in Figure 2. *FAD2A* has two transcript models and *FAD2B* has four transcript models with altered 5′ UTRs, and two transcriptional start sites predicted for each gene. We considered the transcript model AH09G29670.1 for *FAD2A* and AH19G38370.1 for *FAD2B* as a reference for this investigation. Mining of transcriptomic data (http://peanutgr.fafu.edu.cn/index.php) revealed co-expression of both *FAD2* homeologous genes in different tissues including seed, leaf, root tip, root nodule, stem tip, inflorescence etc. (Sinha et al., 2020). FAD2 gene expression was significantly upregulated in peanut seeds, especially during advanced embryo development stages, specifically *FAD2B* had a relatively high expression in root nodules and seed tissues compared to *FAD2A* (Sinha et al., 2020).

In this study, several conserved *cis*-elements important for tissue specific responsiveness to hormonal and environmental cues were observed in the *AhFAD2A* and *AhFAD2B* upstream 5′UTRs (Supplementary File S1). Among them, the *cis*-regulatory RY element (CATGCATG) and 2S seed protein motifs (CAAACAC), which are implicated in seed specific gene expression. The initiator elements (INR) with sequences ‘TTCATTCT’ and ‘TTCATTTT’ responsible for basal transcription and light responsiveness were found. The *cis*-regulatory elements involved in stress response included Anaerobiosis Responsive Element (ARE), Wounding-Responsive Element (WUN motif), W box, G box, Anaeroconsensus among others were also identified. Additionally, the conserved elements for hormonal regulation included Abscisic acid Responsive Element (ABRE), Auxin Responsive Element (AuxRE), GA-responsive elements (GARE motif) etc. were predicted (Supplementary File S1). We chose to focus on the seed specific RY and 2S motifs for this study.

Complete single gRNA CRISPR/Cas9 constructs with a standard or extended gRNA scaffold were developed for the RY and 2S target sites then an *Agrobacterium*-mediated hairy root assay was employed to efficiently test the assembled constructs because traditional peanut transformation and regeneration methods are technically challenging and time consuming. Site-specific mutation frequencies and the type of DNA modifications were analyzed and recorded for each construct through PCR amplification of the target area followed by purification and Sanger sequencing.

**FIGURE 3:**
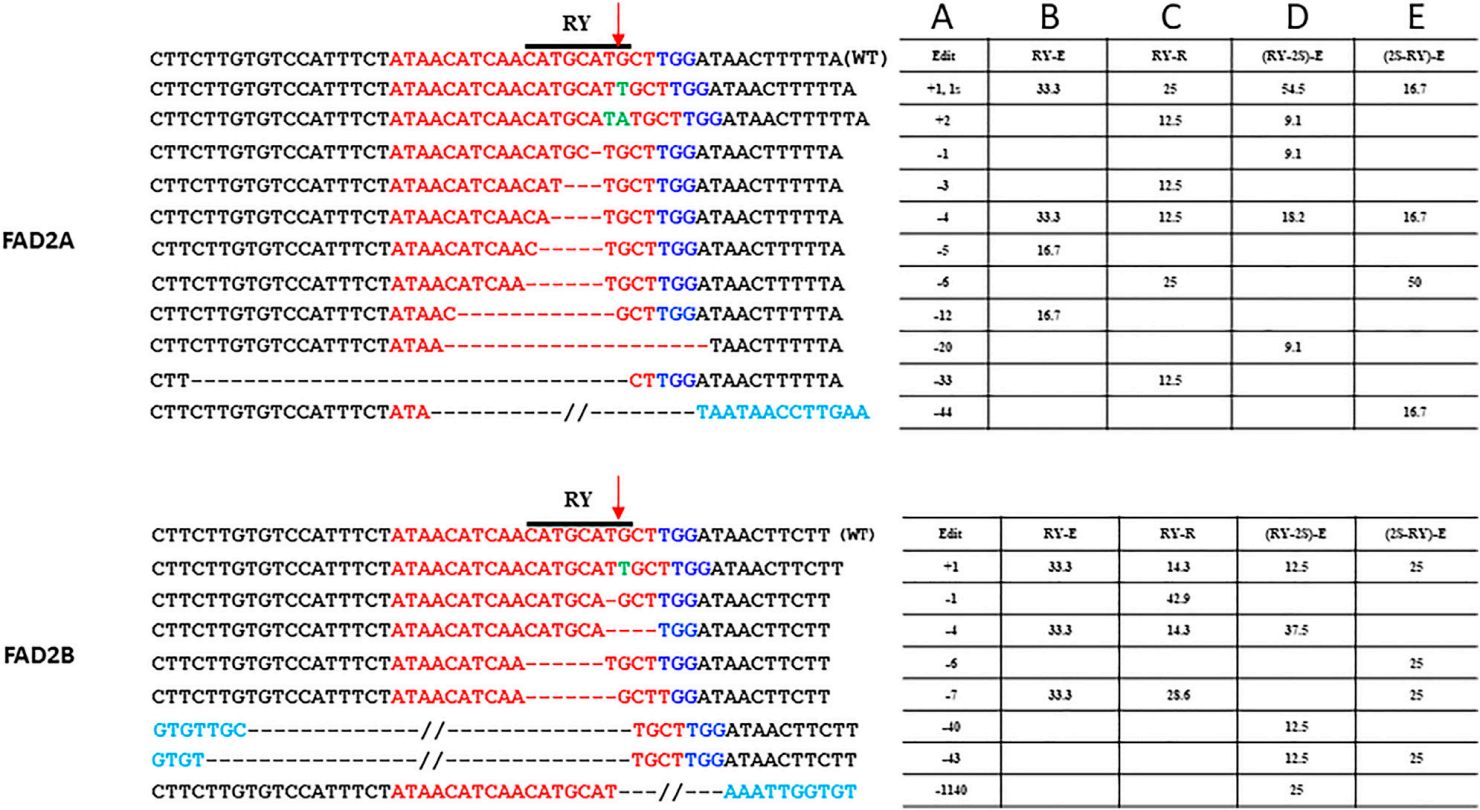
Site-specific modifications at RY repeat element in FAD2A and FAD2B Shown are mutations around the RY motif that were detected for the single RY targeted and duel RY and 2S targeted CRISPR/Cas9 constructs using the hairy root system. The target protospacer sequence is in red, the PAM is in dark blue, insertions are in green, bases in light blue are outside of the wild type sequence that is shown, deletions are represented by dashes, a double slash indicates a deletion larger than what is depicted and the arrow indicated the predicted double strand break site (DSB). Numbers in column A indicate indel size, numbers in columns B to E are percentages of total mutations and the E (extended) or R (standard) designation in columns B to E indicate the gRNA scaffold used.

For the single gRNA constructs, a total of 49 hairy roots for RY and 47 hairy roots for 2S were examined. Indels at the distal RY repeat motif ranged from +2 to −33 bp in *AhFAD2A* and +1 to −7 bp in *AhFAD2B* and the vast majority of edits were indels of less than 10 bp. The extended scaffold produced mutations in *FAD2A* at 20%, *FAD2B* at 10.3% and at both genes 9.68% of the time. The standard scaffold produced mutations in *FAD2A* at 37.5%, *FAD2B* at 50% and at both genes 22.22% of the time (Table 3). Most indels caused a disruption of the RY motif (Figure 3). Based on these figures, the standard gRNA scaffold performed better than the extended gRNA scaffold at RY.

**TABLE 3:** Site-specific editing efficiency in the hairy root assay.

Motif	Construct	Target type	Number of Roots Tested	Number of Edited Roots	FAD2A edits^a^	FAD2B edits^a^	Bi-Homeological edit^b^
RY	Cas9-Ext scaffold	Single gRNA RY	31	6	20%	10.3%	9.68%
		Dual gRNA (RY-2S)	27	9	36.84%	30.77%	18.51%
		Dual gRNA (2S-RY)	20	6	40%	25%	20%
	Cas9-Reg scaffold	Single gRNA RY	18	8	37.5%	50%	22.22%
2S	Cas9-Ext scaffold	Single gRNA 2S	23	2	6.67%	13.33%	4.35%
		Dual gRNA (RY-2S)	27	2	5.26%	3.85%	0
		Dual gRNA (2S-RY)	20	2	0	12.5%	0
	Cas9-Reg scaffold	Single gRNA 2S	24	1	0	4.55%	0

a The percentage of amplicons with edit in total amplicons from FAD2A or FAD2B.

b The percentage of amplicons with both FAD2A and FAD2B edits in total amplicons from FAD2A and FAD2B.

**FIGURE 4:**
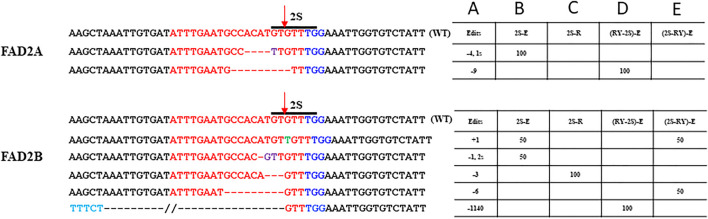
Site–specific modifications at 2S Seed protein element. Shown are mutations around the 2S motif that were detected for the single 2S targeted and duel RY and 2S targeted CRISPR/Cas9 constructs used the hairy root assays. The target protospacer sequence is in red, PAM is in blue, substitutions are in purple, insertions are in green, deletions are represented by dashes and the arrow indicated the predicted double strand break site (DSB). Numbers in column A indicate indel size, numbers in columns B to E are percentages of total mutations and the E (extended) or R (standard) designation in columns B to E indicate the gRNA scaffold used.

The number of mutations detected at the proximal 2S motif were lower than that observed at the RY motif and the type of edits ranged from indels of −4 bp in *FAD2A* and +1 to −3 bp for *FAD2B* with most mutations causing disruption of the 2S motif (Figure 4). The extended scaffold produced mutations in *FAD2A* at 6.67%, *FAD2B* at 13.33% and in both genes at 4.35% of the time. The standard scaffold did not produce detectable mutations in *FAD2A* while 4.55% of mutations were at *FAD2B* (Table 3) Based on these figures, the extended scaffold performed better than the standard gRNA scaffold at 2S.

Dual gRNA editing constructs were generated that target both RY and 2S motifs using the validated targeting oligo sequences used in the single gRNA constructs. These constructs used the extended scaffold because they exhibited improved efficiency overall and were meant to test the efficiency of editing both targets simultaneously, targeting both genes simultaneously the possibility of generating large deletions between RY and 2S. To test for position effect in the gRNA order, two constructs were generated: one with a RY gRNA in the first position and 2S in the second position while the reverse order was used for the second construct.

Analysis of transformed hairy roots showed that overall editing efficiency at RY was greater than at 2S regardless of gRNA order; a result similar to that observed with the single gRNA study. This remained true for edits in both the *FAD2A* and *FAD2B* genes. Most indels at RY were less than 7 bp (80–90% in *FAD2A* and 50–75% in *FAD2B*) and a few (16–25%) deletions were greater than 40 bp (43 bp, 44 bp and 46 bp specifically) (Figure 3). Approximately 18–20% of the lines had RY edits in both *FAD2A* and *FAD2B* using either gRNA order (Table 3). Also noted was a single root containing a large deletion of 1,140 bp between the RY and 2S sites in *FAD2B* (Figure 3).

Using the dual editing constructs, it was noted that editing at the 2S motif was much less effective with only one edit of -9 bp in *FAD2A* when the RY gRNA was in the first position and no edits were observed when 2S was in the first position for *FAD2A*. The opposite was true for *FAD2B* where no edits were observed when the RY gRNA was in the first position, but a +1 edit and a −6 bp edit were observed when the 2S gRNA was in the first position (Figure 4). Additionally, the dual gRNA constructs produced no simultaneous 2S mutations in both *FAD2A* and *FAD2B* (Table 3).

To access the consequences of *FAD2* gene editing on oil quality, *Agrobacterium*-mediated transformation through calyx tube injection was conducted. Single gRNA constructs with the extended scaffold targeting either RY or 2S were used to compare the mutation effect on oil quality at each motif. Approximate 650 calyx tubes were injected with the RY construct strain and 200 of calyx tubes were injected with the 2S construct strain. Following injection, a total of 120 potentially edited seeds were harvested. Among these, 26 seeds had an increased oleic acid content ranging from 55 to 70% when the RY motif was targeted and 7 seeds had an increased oleic acid content ranging from 55 to 60% when the 2S motif was targeted in compared to untransformed seeds with oleic acid concentration of 40–54% (Table 4). The changes of fatty acid in T0 seeds were listed in the Supplementary File S2. Although the apparent transformation efficiency was similar (27.66 RY vs 26.92% 2S) for both constructs, the resulting oleic acid content in the edited RY motif was higher than the edited 2S motif. It should be noted that the oleic acid content of two seeds for the RY motif was 66–70%, while the majority of presumed edits had a lower oleic acid content. Regardless, apparent mutations increased oleic acid concentration in these seed whether the RY or 2S motifs were targeted showing efficacy of this approach. However, amplifications of T_1_ plant DNAs showed no mutations in the targets from either *FAD2A* or *FAD2B*, and all seeds harvested from T_1_ plants derived from these T_0_ edited seeds showed oleic acid content less than 55%. It might indicate that the CRISPR components were delivered into somatic cells rather than germ cells in T0 seeds using the calyx tube injection method.

**TABLE 4:** The content of oleic acid in the T_0_ seeds.

Target		RY				—	2S				—
Oleic Acid Content		40%–54%^a^	55%–60%	61%–65%	—	—	40%–54%	55%–60%	61%–65%	66%–70%	—
Genotype used	AU18-46	32	14			94	8	4	—	—	26
	GT-C20	17	1				7	3	—	—	
	Exp27-1516	19	5	4	2		3	1	—	—	
Total number of seeds harvested		68	26			94	18	8			26
Transformation efficiency (%)		—	—	—	—	27.66^b^	—	—	—	—	26.92

a Same oleic acid content as wild type in some harvested seeds.

b The percentage of number of seeds with increased oleic acid in total number of harvested seeds in each target.

## Discussion

Gene Editing has proven to be a powerful tool for generating gene knockouts for the study of gene function. Although less well studied, promoter editing has been successfully employed to engineer desirable traits such as disease resistance in rice (Oliva et al., 2019) and yield traits in tomato (Rodríguez-Leal et al., 2017). As demonstrated in this study and others, this approach has the potential to generate a wide spectrum of novel transcriptional alleles for genetic fine-tuning and optimized gene expression in crop plants.

The RY repeat motif is implicated in quantitative expression in seeds in diverse species (Baumlein et al., 1992; Lelievre et al., 1992; Dickinson et al., 1998) and is known to interact with B3 domain transcription factors involved in Abscisic acid (ABA) mediated transcription, embryogenesis and seed development (Reidt et al., 2000). Promoter studies by Ellerström et al. (1996) demonstrated that alteration of RY motifs in the *Brassica napus nap A* promoter resulted in changes in tissue specific gene expression. In addition, the 2S seed protein motif is conserved in the promoters of several seed storage protein genes and is implicated in higher activity of the *nap A* promoter (Stålberg et al., 1996). Deletion analysis specifically showed the 2S motif was important in regulation of seed protein accumulation while alterations to the promoter changed gene expression patterns (Stålberg et al., 1993). In this study, we investigated the feasibility and efficiency of simultaneously editing regulatory elements in two related peanut genes. Specifically, the RY and 2S motifs in the *FAD2* genes were altered and an expected increase in oleic acid content of seed was detected suggesting reduced *FAD2* gene expression as a consequence of targeted promoter alteration.

Initial testing of gene editing technology in peanut focused on determining the efficacy of CRISPR/Cas9 technology when single gRNAs were employed. Previously tested vector components were ligated together to create a basic vector platform that contained either a standard or an extended gRNA scaffold then targeting oligos for the RY or 2S motifs were added. Testing of constructs in a hairy root assay demonstrated the following: 1) editing efficiency at the distal RY motif was higher than at the proximal 2S motif, 2) the standard gRNA scaffold was more effective at RY than the extended gRNA scaffold, 3) the extended gRNA scaffold was more effective at 2S than the standard scaffold, 4) *FAD2B* was targeted more efficiently than *FAD2A*, and 5) the predominance of edits involving deletions less than 10 bp with a single gRNA target was consistent with earlier studies (Gisler et al., 2019). Taken together, these data demonstrate the efficacy of CRISPR/Cas9 mediated editing of peanut *FAD2* promoter sequences and suggest that some positions within a promoter may be more accessible than others, use of different gRNA scaffolds may give some flexibility in target efficiency and there may be some difference in homeolog accessibility when targeting gene families.

To further explore the limits of promoter sequence editing, dual gRNA constructs, using the extended gRNA scaffold, were generated to target RY and 2S motifs simultaneously. Two versions of the vector were constructed with RY and 2S targeting oligos in the first and second positions respectively or in the reverse order. The results demonstrated differential editing rates at the target sites with RY being targeted at a higher rate than 2S. Again, *FAD2A* was targeted more frequently than *FAD2B* for the RY motif and *FAD2B* was targeted more frequently than *FAD2A* for the 2S motif. This could be related to the sequence context, local DNA methylation levels, or the chromatin configuration modulating accessibility as suggested by Liu et al., 2019. It should also be noted that this observation was independent of which targeting oligo was in the first position or second position.

Most indels created by the dual gRNA vectors were less than 12 bases while a few were larger. A few longer deletions (>40 bp) at a single site seems to suggest a plausible interaction effect of editing at closely linked sites possibly affecting the local chromatin context. This type of local opening up of chromatin can possibly modulate the timing and activity of the individual gRNAs. This seems analogous to the strategy involving coupling of proximal dead sgRNA (dsgRNA) to the functional sgRNA target, to open up the closed chromatin in rice cells and thereby enhancing editing efficiency (Liu et al., 2019). It should also be noted that one instance of a large deletion (1,140 bp) was detected, which demonstrates that it is possible to generate deletions between to gRNA targets albeit at a low frequency. Taken together this dual gRNA construct data demonstrates that multiple targets can be altered in a gene family in peanut, generating many different types of indels that may be useful for promoter modulation.

With the efficacy of single and dual gRNA constructs demonstrated in peanut, a test of practical application would demonstrate the usefulness of promoter modification. Fatty acids are essential components of plant cells and cell membranes, playing an important role in regulating different abiotic stress responses by modulating cell membrane properties (Iba, 2002; Kachroo and Kachroo, 2009). Thus, the desaturation of fatty acids and the number and position of double bonds in fatty acid chains influences the physical and physiological properties of membranes (Shanklin and Cahoon, 1998; Wu et al., 2009), which impacts proper plant growth and development (Traore and He, 2021). Therefore, general FAD2 loss of function mutations would be predicted to negatively impact cell membranes, leading to poor plant growth and development under abiotic stress conditions. This suggests that existing high oleic acid varieties resulting from mutations in the coding sequences of both *FAD2A and FAD2B*, while commercially useful, may be agriculturally detrimental (Lopez et al., 2000). CRISPR/Cas9 gene editing in peanut may afford a more nuance approach whereby the *FAD2* gene activity in seed could be reduced while maintaining gene activity in other plant tissues.

A calyx tube transformation method was employed using the previously demonstrated RY and 2S single gRNA constructs to test if RY or 2S modification would impact oleic acid content in seed. A total of 120 seeds were generated through this method—94 for the RY construct and 26 for the 2S construct. The transformation efficiency for both constructs was relatively high at 27.66% for the RY construct and 26.92% for the 2S construct based on elevated oleic acid content relative to wild type. When tested by gas chromatograph method, the majority of the seeds had an oleic acid content ranging from 55% to 65%. While none of the 2S seeds tested above 61% oleic acid content, four of the RY edited seed were between 61 and 65% and two seeds were in the range of 66%–70% oleic acid. Taken together, this data suggests that editing of the RY or 2S motifs in the peanut *FAD2* genes can impact oleic acid concentration in seed. Because the increased oleic acid content in T_0_ seeds might be derived from somatic cell mutations, a further experiment will be needed to optimize the parameter for inoculation media, the suitable time and the location of the calyx tube for injection to improve the delivery of construct into reproductive cells with the calyx tube injection method.

Using gene editing to target *cis*-regulatory elements of the *FAD2* gene promoters provides a promising approach to manipulate *FAD2* gene expression in seeds and potentially minimize undesirable pleiotropic effects on other plant tissues while improving the fatty acid profile of seed. The use of CRISPR/Cas9 based promoter or enhancer editing may lead to the development of “cisgenic” plants with optimized *FAD2* gene expression, which may serve as ideal breeding materials for trait introgression, without introducing potentially deleterious alleles and linkage drag, thereby accelerating the pace of cultivar development.

## Data Availability

The datasets presented in this study can be found in online repositories. The names of the repository/repositories and accession number(s) can be found in the article/Supplementary Material.
