# Supplementary material for: CRISPR/Cas9 Based Site-Specific Modification of FAD2 cis-Regulatory Motifs in Peanut (Arachis hypogaea L)
Source: Front Genet. 2022 Apr 27;13:849961. doi: 10.3389/fgene.2022.849961 (PMC9091597; doi:10.3389/fgene.2022.849961)
Supplement: Supplementary file 2 [file Table1.DOCX]

Supplementary file 1. *Cis*-elements in the conserved 5′ UTR intron of FAD2A and FAD2B

FAD2A -327 aaaggaaatgtgtcaacgaattttcaaatatgtccaagagtctctaaaaatagtgctagc -268

FAD2B -324 --gccgcgtctctcacctcgtc---cattgtttt--atcctttggctcgtcgttgcgtcc -272

* * *** * * * * * * * * * *** *

FAD2A ataattgcttaagttaaactgga--------actatttattttacattatgcatagtatg -216

FAD2B cccattgcgttatttcgaaagaagtcaccatcgtatcatttagactttttgtataaaatc -212

***** * * ** * * * *** ** ** ** ** *** **

FAD2A taaaattttttttcagttacttttctatgttattttcttctctcaatttcactagtaaat -156

FAD2B ttgcagtttttgtat--aagatagatacttgcagctctattcatatgaaaattaataatt -154

* * ***** * * * ** * *** ** * * ** *** *

FAD2A atttaaaaattttatttttctattttgtttatcttaacttttggatattgtatttattta -96

FAD2B agttagaactattatgtagatg--gggaatggggtccccgcggggaatggggctccgtgg -96

* *** ** * **** * * * * * * ** ** * * *

FAD2A tctatgaataaaatacaactattatgttaaaaaaaaaaaaaaaacttattagggttgatg -36

FAD2B ggaatgagg-atggggacca-atattcccccacggcggggaatgggggcggggatgggga -38

**** * * * *** * ** ** * *

**+1**

FAD2A ctaaattggtgggttagatcgtcattgatcattgaTCACACCAACTAAAATACCTTAACT +25

FAD2B gcaaatctgagggcgggaatggcagggaggc-------atccccCGCCCCTGCCCTGCCC +16

**** * *** ** * ** ** ** * * ** * *

**+1**

FAD2A TGTTG--TAACTCGAAATATAGCACACTGTTTTCCCTATAAAAACCCAATGTGAGTGAGA +83

FAD2B CATTGACATCCCTAGCCGCGGGTTCGGATAGTATTATATAAAAAC GAGTGAGA +76

*** * * * * ************************

**ABRE**

FAD2A CAACAACTTAACGCATTACATAAAACCTTAAACGTGGCTGCGAGATTCATCATAGGAGAA +143

FAD2B CAACAACTTAACACATTACATAAAACCTTAAACGTGGCTGCGAGATTCATCATAGGAGAA +136

************ ***********************************************

**INR element**

**G Box (Bo)**

**G Box (Ps)**

FAD2A GCACTCACTTCTCTTCTCTCTGTTGGAATTGCTTTCACG**GTTTGCACTATGTTCCTTTAA** +203

FAD2B GCACTCACTTCTCTTCTCTCTGTTGGAATTGCTTTCACG**GTTTGCACTATGTCCCTTTTA** +196

**************************************************** ***** *

FAD2A **TTATAAAACA**-----------------------**TTCTGCTTCTGCTCATTGTATTCTTCT** +240

FAD2B **TTATAAAACTTTCATTATTTCTTTGTTCTGCACTTCTGCTTCTGCTCATTGTATTCTTCT** +256

********* ***************************

FAD2A **ATATAATTCATGCAAATTGCTCTAAAAAATTGAACTCGTGTTGCTGGTTTCTTCTTGTGT** +300

FAD2B **ATATAATTCATGCAAATCGCTCTAAAAAATTGAACTCGTGTTGCTGGTTTCTTCTTGTGT** +316

***************** ******************************************

**NOD consensus**

**RY element**

FAD2A **CCATTTCTATAACATCAACATGCATGCTTGGATAACTTTTTATTTTGATCTTTTATAATA** +360

FAD2B **CCATTTCTATAACATCAACATGCATGCTTGGATAACTTCTTATTTTGATCTTTTATAATA** +376

************************************** *********************

**FAD2A ACCTTGAATTTTTCTGAATTTTGAA-GCAAAGGGGTGAGGTTTTCTTCCATGTTATTTCT +419**

FAD2B **ACCTTGGATTTTCTGAATTCTGAAGTAAGGGTTGGTGAAGTTTTCTTCCATGTTATGTCT** +436

****** ***** * * * * ***** ***************** ***

**WUN motif**

FAD2A **ACTGGATTTCAGATTCTGCATTAAACAATATCAATGAGAATGCTGACAAATTTCTTTTAT** +479

FAD2B **ACTGGATTTCAGATTCTGCATTAAACAATATCAATGAGAATGCTGACAAATTTCTTTTAT** +496

************************************************************

**INR element**

FAD2A **CCTCAGACACACAGTTTTGTTATCCCAGCTGGTTTCATAGCCGTTCGTTTTTCATTTTTC** +539

FAD2B **CCTCAGACACACAGTTTTGTTATCTCAGCTGGTTTCATAGCCGTCCGTTTTTCATTTTTC** +556

************************ ******************* ***************

**Pyrimidine box**

FAD2A **TTTTATTTATTTATTTATTCTTTGGAATCTCTGAAGATTCCCTTTTTTCATTGAACTCCT** +599

FAD2B **TTTTATTTATTTATTTATTCTTTGGAATCTCTGAAGATTCCCTTTTTTCATTGAACTCCT** +616

************************************************************

FAD2A **TCTTGGAAATGACACCTATCATTTATATCTTTACATATAAGACATGAAAATTGTCATATA** +659

FAD2B **TCTTGGAAATGACACCTATCATTTATATCTTTACATATATGACATGAAAATTGTCATATA** +676

*************************************** ********************

**-300 element**

**GARE**

**ARE**

FAD2A **GAAATAGGAATATCTCTTAAACCATAAGATTATTCTGTTGCAAAATGTTTTCTTCTCCCC** +719

FAD2B **GAAATAGGAATATCTCTTAAACCATAAGATTATTCTGTTGCAAAATGTTTTCTTCTCCCC** +736

************************************************************

**Root motif**

**AuxRE**

**SEF4 motif**

FAD2A **ATCACTTTTTCTTTTTAATATTTTTATTTTCTGGCTCCAAGTCCAAGCAATAATTAATGG** +779

FAD2B **ATCACTTTTTCTTTTTAATATTTTTATTTTCTGGCTCCAAGTCCAAGCAATAATTAATGG** +796

************************************************************

**Amylase box**

**Pyrimidine box**

FAD2A **GACCCTTTTTACTTTTTCATGGATAGTAAATATATAAATAAGGAATGTAATGTAATACAA** +839

FAD2B **GACCCTTTTTACTCTTTCATGGATAGTAAATATATAAATAAGGAATGTAATGTAATACAA** +856

************* **********************************************

**NOD consensus**

**NOD consensus**

FAD2A **AAACATTACAACTTCTCTTCTTGGAGTATAGTTGCATTT**-**TTTTCATCCATAATCTTTTC** +898

FAD2B **AAACACCACAACTTCTCTTTTTGGAGTATAGTTGCAATTTTTTTTATCCATAATCTTTTC** +916

***** ************ **************** ** **** ***************

**W box**

**Root motif**

FAD2A **CTTGAGGTTAATCTTCTTTTCTAATTTGCCTTGAAAATGACTGAATTATATTTGATTTTA** +958

FAD2B **CTTGAGGTTAATATTCTTTTCTAATTTGCCTTGTAAATGACTGAATTATATTTGATTTTA** +976

************ ******************** **************************

**ARE**

FAD2A **CTTTTTCTGTTAGTATGAATTACTAAACCAGAAATCCATTGCCATTTCCTTTAGTAGTAT** +1018

FAD2B **TTTTTTCTGTTAGTATGAATTACTAAACCAGAAATCCATTGCCATTTCCTTTAGTAGTAT** +1036

***********************************************************

FAD2A **AATTCATTCTGGTTGAAGAGAAAAATTAGAAGAAAATATTTGCATACATTAAGGCATTAG** +1078

**INR element**

**Root motif**

FAD2B **AATTCATTCTGGTTGAAGAGAAAAATTAGAAGAAAATATTTGCATACATTTAGGCATTAG** +1096

************************************************** *********

FAD2A **TTTTGATGGCATGTTTGCAAAAAATTGATGTATCATAATTGCTTCTGGGCCCTCACTGAT** +1138

FAD2B -----------------------------------**TAATTGCTTCTGGGCCCTCACTGAT** +1121

*************************

**2S seed protein motif**

FAD2A **ATATGATTTTGTCATTGTGTATTTGTGTTTGGTTTATGATAGTTTCTGTTTTTGCATGTC** +1198

FAD2B **ATATGATTTTGTCATTGTGTATTTGTGTTTGGTTTATGATAGT**------**TTTTGCATGTC** +1175

******************************************* ***********

FAD2A **AACCTCACACGTATACTAATACAAGTGAACCTCAGAATCATGCCCCTTACAACTATAT**-T +1257

FAD2B **AACCTCACACGTATACTAATACAAGTGAACCTCAGAATCATGCCCCTTACAACTATATAT** +1235

********************************************************** *

FAD2A **ATAGTGTGAAATTAATGTTTCTGTGCATGTGTCATGTGAGTCTGTGACACTGCTACCACC** +1317

FAD2B **ATAGTGTGAAATTAATGTTTCTGTGCATGTGTCATGTGAGTCTGTGACACTGCTACCACC** +1295

************************************************************

FAD2A **ATCCAAAATCCAAAGAAGTTGTGTGTGTTGTAGTGGTTAACCCTTGACACACTCTTCATG** +1367

FAD2B **ATCCAAAATCCAA**-**ACAGTTGTGTGTGTTGTAGTGGTTAACCCCTGGCACACTCTTCAAG** +1354

************* *************************** ** *********** *

FAD2A **CTCTTTTGCTCTGTCCTTCATTATAGAAGATGCATGCTTTCTTGTCAATTGAAGGGTGCA** +1427

FAD2B **CTCTTTTGCTCTGTCCCTCATTATAGAAGATGCATGCTTTCTTGTCAAATGAAGGGTGCA** +1414

**************** ******************************* ***********

**Anaero consensus**

**Root motif**

FAD2A **AAACATCATCACTCATACTAGTCACTTGGCATGATAAGCTAAATTGTGATATTTGAATGC** +1487

FAD2B **AAACATCATCACTCATACTAGTCACTTGGCATGATAAGCTAAATTGTGATATTTGAATGC** +1474

************************************************************

**2S seed protein motif**

FAD2A **CACATGTGTTTGGAAATTGGTGTCTATTGTGTTATGCATTTTCACATCCTTTCTTTTCTT** +1547

FAD2B **CACATGTGTTTGGAAATTGGTGTCTATTGTGTTAGGCATTTTCACATCCTTTCTTTTCTT** +1534

********************************** *************************

**SEF4 motif**

FAD2A **TTCTCTATTTTTATAGGATTCCCAAGGCATAATAGAACTTCAAATAACTGGTTGTAATAT** +1607

FAD2B **TTCTCTATTTTTATAGGATTCCCAAGGCATAATAGAACTTCAAATAACTGGTTGTAATAT** +1594

************************************************************

FAD2A **CAGAAATCCTTTCTTTGCTGTTTTTCTGGTCATGGTCTAAGTCACTCTCATCTGCAATGA** +1667

FAD2B **CAGAAATCCTTTCTTTGCTGTTTTTCTGGTCATGGTTTAAGTCACTCTCATCTGCAATGA** +1654

************************************ ***********************

FAD2A **CTATCATTCATTCATTTTCTTAGATATCAGAATCATTAGATTACTGATTATTGACTTGCT** +1727

FAD2B **CTATCATTCATTCATTTTCTTAGATATCAGAACCATTAG**-------------------**CT** +1695

******************************** ****** *

**Prolamin box**

FAD2A **TTGTAGTAGTGCAAAGTGCTAACTCTTTCTTTCTACATTGGTAACAG**GAGCTTTAACAAC +1787

FAD2B **TTGTAGTAGTGCAAAGTGCTAACTCTTTCTTTCTACATTGGTAACAG**GAGCTTTAACAAC +1755

************************************************************

**ORF Start**

FAD2A ACAACA*ATGGGAGCTGGAGGGCGTGTCACTAAGATTGAAGCTCAAAAGAAGCCTCTTTCA* +1847

FAD2B ACAACA*ATGGGAGCTGGAGGGCGTGTCACTAAGATTGAAGCTCAAAAGAAGCCTCTTTCA* +1815

************************************************************

The alignment of upstream sequence, 5’ UTR, intron and part of the coding sequence of reference transcript 1 of FAD2A and FAD2B genes, respectively, are shown. The transcriptional initiation (+1, blue highlight) and translational initiation site (ORF Start, green highlight) are marked. The intron in the 5’UTR is shown in bold and the conserved putative regulatory motifs are boxed. The motifs targeted for modification are indicated as colored boxes. The potential TATA box (light grey) and CAAT box (dark grey) are highlighted. The upstream sequences are indicated in lower case letters, exon in the 5’UTR is depicted in upper case, ORF is in italics.
